# Neuroprotective and Immunomodulatory Action of the Endocannabinoid System under Neuroinflammation

**DOI:** 10.3390/ijms22115431

**Published:** 2021-05-21

**Authors:** Ludmila A. Kasatkina, Sonja Rittchen, Eva M. Sturm

**Affiliations:** 1 Otto Loewi Research Center, Division of Pharmacology, Medical University of Graz, 8010 Graz, Austria; ludmilka.kasatkina@gmail.com (L.A.K.); sonja.rittchen@medunigraz.at (S.R.); 2 Department of Anatomy and Structural Biology, Albert Einstein College of Medicine, Bronx, NY 10461, USA

**Keywords:** endocannabinoids, N-acylethanolamines, neuroinflammation, glutamate-mediated excitotoxicity, neurodegenerative diseases, synaptic plasticity

## Abstract

Endocannabinoids (eCBs) are lipid-based retrograde messengers with a relatively short half-life that are produced endogenously and, upon binding to the primary cannabinoid receptors CB_1/2_, mediate multiple mechanisms of intercellular communication within the body. Endocannabinoid signaling is implicated in brain development, memory formation, learning, mood, anxiety, depression, feeding behavior, analgesia, and drug addiction. It is now recognized that the endocannabinoid system mediates not only neuronal communications but also governs the crosstalk between neurons, glia, and immune cells, and thus represents an important player within the neuroimmune interface. Generation of primary endocannabinoids is accompanied by the production of their congeners, the N-acylethanolamines (NAEs), which together with N-acylneurotransmitters, lipoamino acids and primary fatty acid amides comprise expanded endocannabinoid/endovanilloid signaling systems. Most of these compounds do not bind CB_1/2_, but signal via several other pathways involving the transient receptor potential cation channel subfamily V member 1 (TRPV1), peroxisome proliferator-activated receptor (PPAR)-α and non-cannabinoid G-protein coupled receptors (GPRs) to mediate anti-inflammatory, immunomodulatory and neuroprotective activities. In vivo generation of the cannabinoid compounds is triggered by physiological and pathological stimuli and, specifically in the brain, mediates fine regulation of synaptic strength, neuroprotection, and resolution of neuroinflammation. Here, we review the role of the endocannabinoid system in intrinsic neuroprotective mechanisms and its therapeutic potential for the treatment of neuroinflammation and associated synaptopathy.

## 1. Highlights

- Retrograde endocannabinoid signaling provides a mechanism by which neurons can rapidly regulate the strength of their synaptic inputs.
- Stimulation of postsynaptic neurotransmitter receptors and sustained Ca^2+^ influx is a potent trigger for the production of endocannabinoids (eCBs) and their congeners.
- Neuroinflammation and alterations in endocannabinoid signaling is implicated in multiple neurological disorders.
- The activity-dependent flow of glutamate and eCBs from synapses controls microglial attraction, secretion of pro-inflammatory and pro-survival factors, and defines the synapse stability under inflammation and excitotoxicity.
- The pharmacological inhibition of eCB degradation exerts a primary effect in injured sites, where these mediators are actively produced de novo.
- The endocannabinoid system mediates communication within the tripartite synapse during the development and resolution of neuroinflammation.

## 2. Introduction

Neuroinflammation is widely regarded as inflammation of the central nervous system (CNS) comprising the brain and spinal cord. The inflammatory process is driven by the release of pro-inflammatory mediators such as cytokines, prostaglandins, and reactive oxygen and nitrogen species by activated endothelial and glial cells with subsequent infiltration of peripheral inflammatory cells into the CNS. As a consequence, neuroinflammation can lead to edema, tissue damage, and loss of neuronal functions as well as accelerate and cause cognitive impairment and neurodegenerative diseases. Common triggers of chronic neuroinflammation include toxic metabolites, harmful self-proteins during autoimmunity, aging, bacterial and viral infections, as well as traumatic brain, and spinal cord injury.

The endocannabinoid system (ECS), consisting of the cannabinoid receptors CB_1_ and CB_2_, their major endogenous ligands 2-arachidonylglycerol (2-AG) and arachidonoylethanolamide (AEA or anandamide), and their synthesizing and degrading enzymes, plays a crucial role in the intrinsic response to neuroinflammation, brain injury, and neurodegenerative diseases [1,2,3]. This system is now being expanded with the ligands that do not show affinity for CB_1/2_ but display cannabimimetic activity and are able to modulate the actions of true eCBs. N-acylethaolamines generated as AEA congeners together with lipoaminoacids and acyl conjugates of neurotransmitters exert their biological activities through different receptors such as peroxisome proliferator-activated receptor (PPAR)-α [4], transient receptor potential cation channel subfamily V member 1 (TRPV1) and non-cannabinoid G-protein coupled receptors (GPRs). These compounds as well as true eCBs, their molecular targets, overall interplay, and metabolism comprise the endocannabinoidome having profound role in homeostatic response to noxious endogenous and exogenous stimuli.

The existence of multiple targets and routes for ligand synthesis and degradation within the endocannabinoidome enables complex scanning of cellular states and allows a tightly regulated response to relevant changes. Of particular interest is the involvement of this system in inherent protective responses against inflammatory and neurodegenerative processes in the brain as targeted manipulation of this system has high therapeutic potential. In this review, we provide an overview of the immune-regulatory and neuroprotective potential of the endocannabinoidome during neuroinflammation.

## 3. The Endocannabinoid System

The ECS is a complex biological network involved in the maintenance of homeostasis. It consists of a family of naturally occurring signaling lipids, the endocannabinoids (eCBs), their synthesizing and degrading enzymes, specific transmembrane eCB transporters, and receptors. Components of the ECS are found throughout the body: in the CNS, in cells of the immune system, the liver, the reproductive system, the respiratory system, the gastrointestinal tract, the cardiovascular system, and in skeletal muscles.

### 3.1. Metabolism of Endocannabinoids and Related Compounds

**Figure 1:**
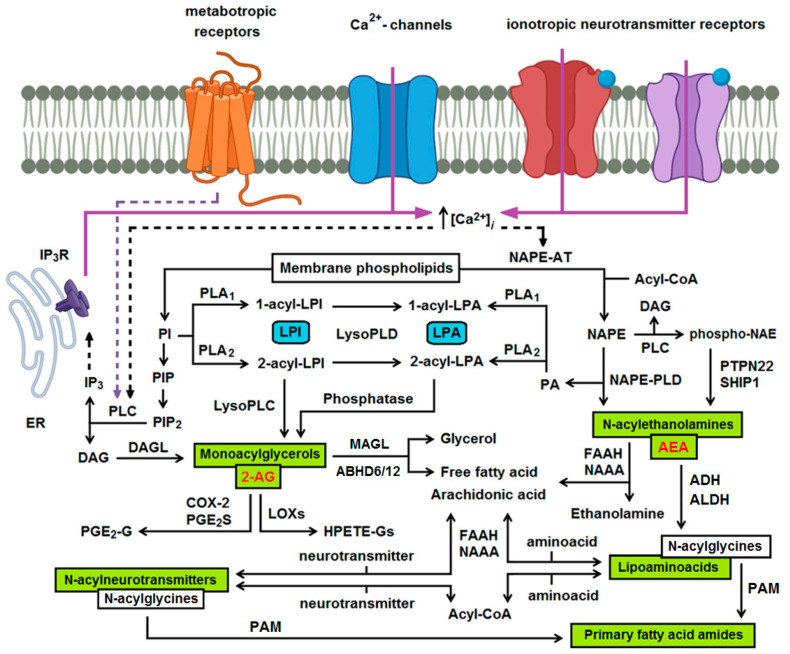
Main pathways of endocannabinoid (eCB) generation and degradation. Monoacylglycerols, such as 2—arachidonylglycerol (2-AG) and N-acylethanolamines such as arachidonoylethanolamide (AEA) are generated from membrane phospholipids and provide precursors used in the synthesis of lipoaminoacids and N-acylneurotransmitters. Production of eCBs is associated with the generation of lysophospholipids, like lysophosphatidylinositol (LPI) and lysophosphatidic acid (LPA). ABHD6/12, alpha/beta-hydrolase domain containing 6/12; ADH, alcohol dehydrogenase; 2-AG, 2-arachidonylglycerol; ALDH, aldehyde dehydrogenase; COX, cyclooxygenase; DAG, diacylglycerol; DAGL, diacylglycerol lipase; FAAH, fatty acid amide hydrolase; HPETE-Gs, glycerol esters of 12- or 15-hydroperoxyeicosatetraenoic acid; IP_3_R, inositol trisphosphate receptor; LOXs, lipoxygenases; MAGL, monoacylglycerol lipase; NAAA, NAE-hydrolyzing acid amidase; NAE, N-acylethanolamine, NAPE, N-acyl-phosphatidylethanolamine; NAPE-AT, NAPE forming N-acyltransferase; PA, phosphatidic acid; PAM, peptidyl-glycine alpha-amidating monooxygenase; PGE_2_-G, prostaglandin E_2_–glycerol ester; PGE_2_S, prostaglandin E_2_ synthase; PI, phosphatidylinositol; PIP, phosphatidylinositol 3-phosphate; PIP_2_, phosphatidylinositol 4,5-bisphosphate; PL, phospholipase; PTPN22, protein tyrosine phosphatase non-receptor type 22; SHIP1, SH-2 containing inositol 5′ polyphosphatase 1.

Levels of eCBs vary among specific regions of the brain and differ between animal species. In rat brain, basal 2-AG levels were estimated at 2–10 nmol/g [5], whereas AEA was detected at the range of 3–30 pmol/g [6] and typically comprises only 1–3% of generated NAEs [7]. Although both, 2-AG and AEA, are referred to as eCBs their metabolic pathways are completely different. 2-AG is formed from arachidonic acid-containing membrane phospholipids via three major pathways: * from diacylglycerol (DAG) via DAG lipase, ** from 2-acyl lysophosphatidic acid (LPA) via 2-LPA phosphatase, and *** from 2-acyl lysophosphatidylinositol (LPI) via lyso-phospholipase C. Similarly, AEA and related NAEs are produced on demand in response to certain stimuli [8,9] from membrane glycerophospholipids via the transacylation–phosphodiesterase pathway comprising two main enzymes, Ca^2+^-dependent N-acyltransferase and N-acyl-phosphatidylethanolamine-hydrolyzing phospholipase D (NAPE-PLD) (Figure 1). Lysophospholipids, generated in the course of 2-AG synthesis and produced at higher levels under inflammatory conditions such as neuroinflammation [10], possess biological activity themselves. For instance, LPI and its metabolite LPA activate respectively GPR55, GPR119, and LPA receptors to modulate cell proliferation, migration, cytokine and chemokine secretion, apoptosis/survival, and tumorigenesis.

The major degradation pathway of 2-AG is considered to be the hydrolysis to arachidonic acid and glycerol, which can be catalyzed by multiple enzymes, including monoacylglycerol lipase (MAGL), fatty acid amide hydrolase (FAAH), α/β-hydrolase domain containing (ABHD) 6, and ABHD12 proteins (reviewed in [4,11]). Furthermore, the arachidonoyl moiety of 2-AG can be oxygenated by cyclooxygenase (COX)-2 and lipoxygenases, resulting in the formation of pro-inflammatory glyceryl prostaglandins, such as prostaglandin E_2_–glycerol ester (PGE_2_-G), or hydroperoxy derivatives of 2-AG, respectively [12,13]. Although physiological significance of the oxygenation pathways remains unclear, biological activities of glyceryl prostaglandins have been reported [12]. The essential degradation pathway of NAEs is their hydrolysis to free fatty acids and ethanolamine by FAAH. Alternatively, NAE-hydrolyzing acid amidase (NAAA), a lysosomal enzyme, that is structurally similar to acid ceramidase and hydrolyzes NAEs at acidic pH, was described [14]. Both, specific FAAH and NAAA inhibitors, represent promising therapeutic tools for the treatment of neurological and inflammatory disorders as they raise the endogenous levels of bioactive NAEs [15,16]. In addition to hydrolytic degradation, polyunsaturated NAEs like AEA, DHEA, and EPEA are oxygenated by COX-2, lipoxygenases and cytochrome P450s and converted into prostaglandin ethanolamides (prostamides) or ω-3 endocannabinoid epoxides, which exhibit unique biological activities themselves [17,18]. The synthesis and degradation pathways of eCBs are summarized in Figure 1.

Lipoaminoacids and N-acylneurotransmitters are synthesized as a result of the conjugation of an amino acid/neurotransmitter with free fatty acids or acyl-CoA. Additionally, N-arachidonoylglycine can be generated from AEA in sequential steps catalyzed by alcohol dehydrogenase and aldehyde dehydrogenase. N-arachidonoyldopamine (NADA) biosynthesis in brain occurs through direct conjugation of dopamine with arachidonic acid. In this process FAAH is either a rate-limiting enzyme that liberates arachidonic acid from AEA, a conjugation enzyme, or both [19]. N-acylneurotransmitters and lipoaminoacids are degraded by FAAH or through modification of acyl- or amino acid/neurotransmitter residues.

### 3.2. Molecular Targets of the Endocannabinoid System

The main eCBs 2-AG and AEA bind to the central and peripheral G protein-coupled cannabinoid receptors CB_1_ and CB_2_. 2-AG behaves as a full agonist at CB_1_ and CB_2_, while AEA is a partial agonist of CB_1_, but almost inactive at CB_2_. Both CB_1_ and CB_2_ are G protein coupled receptors that have the ability to simultaneously activate multiple pools of G proteins. First, both receptors were shown to be associated with G proteins of the G_i/o_ family, while later additional signaling via G_s_ and G_q_ has been reported. G_i/o_ subunits inhibit adenylyl cyclase or couple to the mitogen-activated protein kinase (MAPK) pathway [20,21] G_s_ stimulates adenylyl cyclase [22], G_q_ couples to phospholipase C to promote the release of intracellular calcium [23,24] and G_βγ_ subunits derived from G_i/o_ inhibit voltage-gated calcium channels (VGCC) [25]. Further, coupling of arrestins with both CB_1_ and CB_2_ is implicated in the receptor desensitization, internalization, and G protein–independent signaling [26,27,28].

CB_1_ receptors are expressed predominantly in the nervous system, with enrichment in GABAergic axon terminals, and are among the most abundant GPCR in the brain, whereas CB_2_ receptors are mainly expressed in cells of the immune system and also detected in microglia. The neuronal expression of CB_2_ receptors, which for a long time remained controversial, is now shown on brainstem neurons and midbrain dopaminergic neurons [29,30,31]. The important features of neuronal CB_2_ are their low basal expression compared to CB_1_, and high inducibility under relevant stimuli like inflammation or addiction [32,33]. The functional contribution of CB_1/2_ (as receptors of true eCBs, 2-AG and AEA) in different brain cells is shown in Table 1.

**Figure 2:**
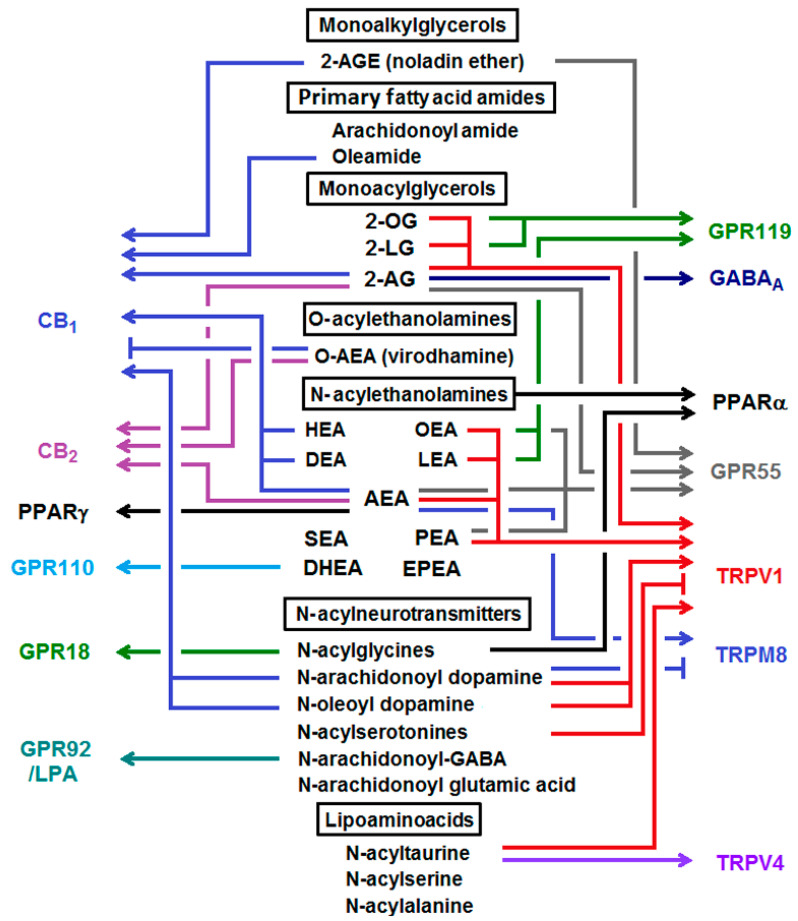
The endocannabinoids(eCBs), related compounds and their molecular targets. Major eCBs are monoacylglycerols (such as 2-arachidonylglycerol), N-acylethanolamines (such as arachidonoylethanolamide), lipoaminoacids, N-acylneurotransmitters or primary fatty acid amides, and have different affinity for cannabinoid receptors (CB)_1/2_, non-cannabinoid G-protein coupled receptors (GPRs), and transient receptor potential cation channel subfamily V member 1 and 4 (TRPV1 and TRPV4). 2-AGE, 2-arachidonyl glyeryl ether (nolandin ether); DEA, docosatetraenoylethanolamide; DHEA, docosahexaenoylethanolamide; EPEA, eicosapentaenoylethanolamide; HEA, homo-γ-linolenylethanolamide; LEA, linoleylethanolamide; 2-LG, 2-linoleoylglycerol; LPA5, lysophosphatidic acid receptor 5; OEA, oleoylethanolamide; 2-OG, 2-oleoylglycerol, PEA, palmitoylethanolamide; SEA, stearoylethanolamide; TRPM8, transient receptor potential cation channel subfamily M (melastatin) member 8.

Besides CB_1_ and CB_2_, a range of NAEs and monoacylglycerols also interact with several other receptors (Figure 2). AEA activates the transient receptor potential cation channel subfamily V member 1 (TRPV1), implicated in synaptic transmission and pain sensation, and seems to be a partial agonist for the non-CB receptor GPR55 [35]. Moreover, the interaction of 2-AG and non-CB receptors has emerged recently. Several studies have suggested that in addition to CB_1_ and CB_2_, there are non-CB_1_ and non-CB_2_ cannabinoid-related orphan GPCRs including GPR18, GPR55, and GPR119 [36]. It was demonstrated that 2-AG binds to GABA_A_ receptors and can modulate the action of neurosteroids at GABA_A_ receptors [37]. Moreover, endocannabinoids and some of their metabolites bind and activate peroxisome proliferator-activated receptors (PPARs) [38].

**Figure 3:**
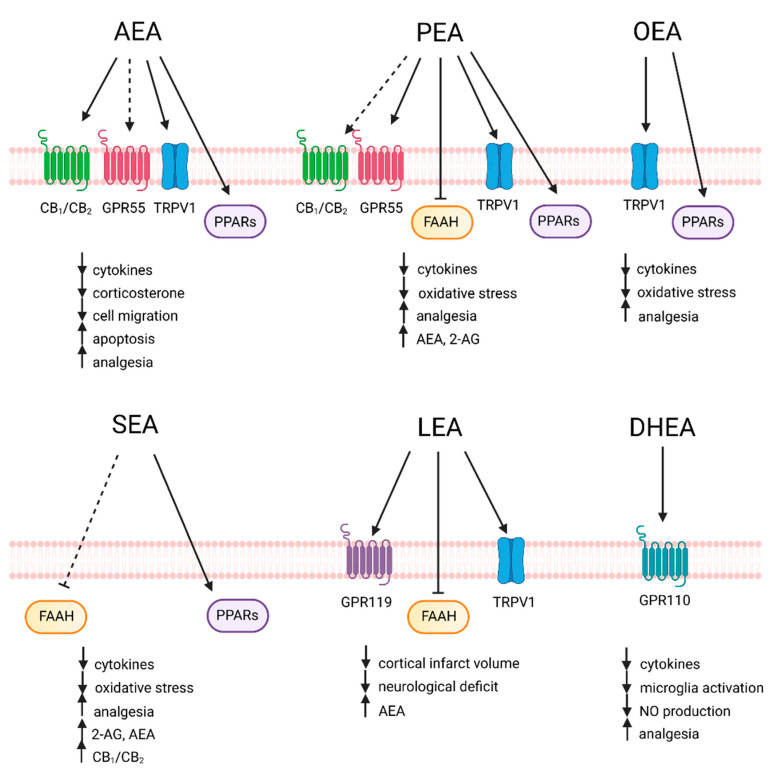
Signaling pathway and anti-inflammatory actions of NAEs. Arachidonoylethanolamide (AEA) signals via the cannabinoid receptors CB_1/2_, the non-cannabinoid G protein coupled receptor (GPR)55, the transient receptor potential cation channel subfamily V member 1 (TRPV1), and the peroxisome proliferator-activated receptor (PPAR)-α and γ. Palmitoylethanolamide (PEA) has been shown to inhibit the fatty acid amide hydrolase (FAAH) and to signal via GPR55, TRPV1, and PPAR-α, while CB_1/2_binding is still controversial. Oleoylethanolamide (OEA) activates TRPV1 and PPAR-α, while stearoylethanolamide (SEA) inhibits FAAH and activates PPAR-α. Linoleylethanolamide (LEA) was shown to activate TRPV1, GPR119, and to inhibit FAAH, while docosahexaenoylethanolamide (DHEA) signaling involves the activation of GPR110.

Saturated and monounsaturated NAEs do not show ligand affinity for cannabinoid receptors, but rather exert their biological activities through different receptors and pathways. PEA (C16:0 *N*-acylethanolamine) has been shown to promote anti-inflammatory, analgesic, anti-oxidative, and neuroprotective actions [39,40]. Most of its effects are mediated by PPAR-α [41]. Moreover, it has been reported that PEA activates TRPV1 [42] and GPR55 [43], and enhances 2-AG and AEA levels via inhibition of FAAH expression [44]. OEA (C18:1 *N*-acylethanolamine) is mainly known for its anorexic activity in experimental models, but has also been shown to mediate anti-inflammatory, analgesic, and antioxidative effects. OEA binds with high affinity to PPAR-α [45]) and interacts with TRPV1 [46]. Recent evidence supports the assumption that SEA (C18:0 *N*-acylethanolamine) mediates anti-inflammatory and neuroprotective activities [47], although there is no direct data on its molecular targets. In the tetrad of behavioral tests considered to be highly predictive for cannabimimetic compounds, SEA behaves similarly to AEA. This may be a result of so-called “entourage” effect of SEA, which potentiates the effects of AEA by inhibiting its degradation [48]. SEA demonstrated neuroprotective properties, decreased the onset of inflammation, and restricted leukocyte infiltration into the brain parenchyma in a mouse model of systemic inflammation. It seems that there is a certain therapeutic window for SEA treatment following the onset of inflammation. Mice treated with SEA had higher levels of 2-AG or its stable isomer 1-AG, in the prefrontal cortex and hippocampus. This rise of 2-AG/1-AG levels preceded the increase of neuronal CB_1/2_ expression and suggests the interference of SEA with the eCB system [47]. Moreover, SEA has been shown to protect from oxidative stress and to have analgesic properties. Similar to AEA, PEA, and OEA, it was demonstrated that SEA, together with linoleylethanolamide (LEA), eicosapentanoylethanolamide (EPEA), and docosahexaenoylethanolamine (DHEA) are activators of PPAR-α [49]. LEA was shown to activate TRPV1 [50], GPR119, and to inhibit AEA hydrolysis by FAAH. Pretreatment with LEA prior to ischemia/reperfusion injury significantly reduced cortical infarct volume and neurological deficit [51]. N-docosahexaenoylethanolamide (DHEA, synaptamide) promotes neurogenesis, neuritogenesis, and synaptogenesis [52] as an endogenous ligand of GPR110 [53], a member of the adhesion-GPR family which is highly expressed in fetal brain. Under systemic inflammation, immune-regulatory functions of GPR110 contribute to the anti-inflammatory action of synaptamide [54]. A summary of the known signaling pathways and anti-inflammatory actions of the major endogenously produced NAEs is presented in Figure 3.

N-acylneurotransmitters and lipoaminoacids represent a separate cluster within the endocannabinoidome, that bears the potential for identification of novel receptor targets in this system. Among these compounds N-acyldopamines are CB_1_ and TRPV1 agonists, while N-acylserotonines are TRPV1 antagonists and N-arachidonoyl-γ-aminobutyric acid (NAGABA) activates GPR92 receptor. Liberation of free neurotransmitter following degradation of N-acylneurotransmitters makes their action more complex.

### 3.3. Involvement of Endocannabinoid System in Response to Neuropathology

Endocannabinoids and related NAEs are produced on demand and play a crucial regulatory role in metabolic processes, behavior, and immunity. Under healthy conditions these lipid mediators are most abundant in the brain and barely found in circulation and peripheral tissues [55]. During various pathological conditions of the CNS the profiles of eCBs and their congeners undergo significant changes, which is associated with the inflammation-modulating, analgesic, and neuroprotective activity of these compounds (summarized in Table 2).

## 4. Glutamate Receptor-Mediated Neurotoxicity

### 4.1. Glutamate as a Major Excitatory Neurotransmitter in Mammals and Potential Neurotoxin

Activation of postsynaptic neurotransmitter receptors and Ca^2+^ influx into the postsynaptic terminal induce the synthesis of eCBs and related compounds. This activity-dependent production of eCBs is essential for the fine regulation of neurotransmission. In the mammalian brain, glutamate is the main excitatory neurotransmitter implicated in learning and memory formation. Glutamatergic neurotransmission mediates synaptic plasticity, whereby ionotropic and metabotropic (mGluRs) glutamate receptors play a primary role. Between the quantal neurotransmitter releases, the level of glutamate in the synaptic cleft is estimated to be <1 μM. This low basal level is maintained by rapid reuptake of glutamate from the extracellular space into the cytosol by high-affinity glutamate transporters EAATs (excitatory amino acid transporters). EAATs are localized on neurons (primarily EAAT4 and EAAT3 (EAAC1, Excitatory Amino Acid Carrier)) and astrocytes (primarily glutamate transporter GLT-1 and glutamate-aspartate transporter GLAST), and co-transport one molecule of L-glutamate (or L-/D-aspartate) with 3Na^+^ and 1H^+^ in exchange of 1K^+^ [66]. The dependence of this transport system on Na^+^ and K^+^ gradients across the plasma membrane makes it highly vulnerable to ATP depletion with subsequent inhibition of glutamate uptake or reversal of transporters [67]. Another factor affecting the efficiency of glutamate removal from the synaptic cleft is translational control of EAATs or post-translational modification of the transporter molecules, which affects the level of active transporters. Glutamate is further accumulated in the synaptic vesicles via vesicular glutamate transporters VGLUTs using the Δμ_H_^+^ gradient.

Considering the high energy-dependent compartmentalization of glutamate and its gradient across the synaptic bouton, i.e., from synaptic vesicles (~200 mM [68]) to the synaptic cleft (<1 μM between release events, around 1 mM during the peak of SV release [69]), any factors affecting the efficiency of high-affinity glutamate uptake represent a potential risk of neurotoxic neuronal damage. The pathophysiological conditions underlying the long-term glutamate rise in the synaptic cleft and extrasynaptic glutamate spillover are traumatic brain injury, ischemia, and other causes of hypoxia, stroke, and oxidative stress leading to transition of the significant portions of EAATs to the reverse mode, when glutamate is released from the cytosol to the extracellular space. Glutamate-mediated neurotoxicity originates from overstimulation of ionotropic glutamate receptors, primarily N-methyl-D-aspartate (NMDA) receptors, and massive Ca^2+^ flux to the postsynaptic terminal. High extracellular concentrations of glutamate lead to the prolonged co-activation of synaptic and extrasynaptic (localized to non-synaptic sites) NMDA receptors, glutamate-mediated neurotoxicity [70], and are involved in the pathogenesis of Alzheimer’s disease, amyotrophic lateral sclerosis (ALS), and Huntington’s disease. 

Due to Ca^2+^ permeability and high affinity for glutamate, NMDA receptors are among the primary molecular targets implicated in the pathogenesis of excitotoxicity. At resting membrane potential, the current through channels of NMDA receptors is almost fully blocked by Mg^2+^ preventing the conductance between the stimuli. During quantal neurotransmitter release two conditions for Ca^2+^ influx through NMDA receptors are met: * glutamate concentrations rise rapidly, and ** the depolarization of synaptic membranes removes the Mg^2+^ block from NMDA receptor channels. Therapeutic concentrations (1–10 μM) of the NMDA receptor antagonist memantine, used for treatment of Alzheimer’s disease, preferentially block extrasynaptic rather than synaptic currents through NMDA receptors in the same neuron [71]. The mode of memantine action enables effective prevention of excessive extrasynaptic NMDA receptor stimulation, with much less effect on NMDA receptor-mediated synaptic activity, when glutamate is elevated for only milliseconds [72].

Under prominent rise of intracellular Ca^2+^ levels, vesicular glutamate release is another factor contributing to elevated extracellular glutamate concentration and excitotoxic damage. In rats and mice, ischemic conditions, followed by release of axonal vesicular glutamate into the peri-axonal space under the myelin sheath, trigger activation of myelinic GluN2C/D-containing NMDA receptors [73], which are generally extrasynaptic [74].

### 4.2. Excitotoxicity as a Prerequisite and Consequence of Neuroinflammation and Neurodegeneration

Due to the ability of Ca^2+^ to activate a range of enzymes, glutamate receptor-mediated excitotoxicity provokes necrotic and apoptotic neuronal death. Massive influx of Ca^2+^ overloads the intracellular buffer systems for this ion, provokes mitochondrial dysfunction, and activation of a range of proteases, including caspases and calpain, leading to the subsequent degradation of components of the neuronal cytoskeleton and the release of apoptotic factors. 

**Figure 4:**
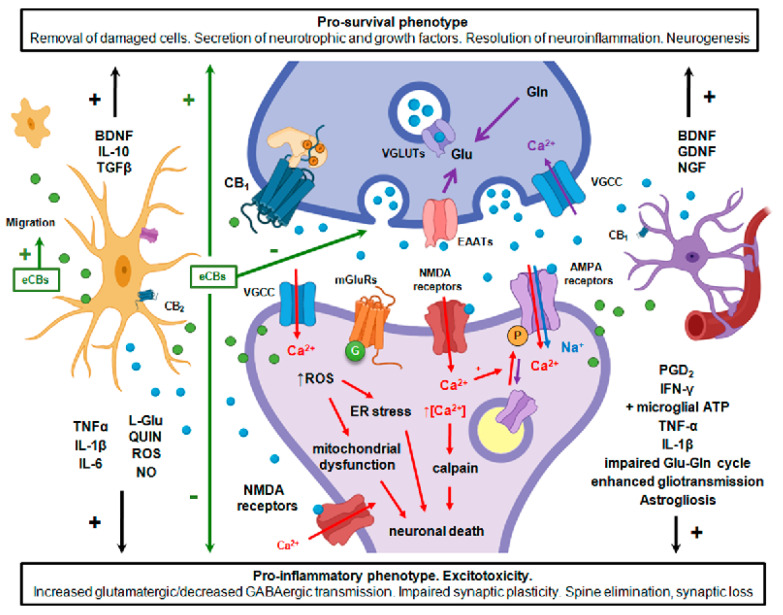
The development of synaptic dysfunction and the involvement of the ECS in neuroprotective responses. Endocannabinoid produced in the glutamatergic synapse under excitotoxic conditions attract microglial cells into close proximity to spines. By adopting a pro-inflammatory or pro-survival phenotype microglia largely define the fate of the injured cells and spines. eCB signaling decrease the presynaptic neurotransmitter release, support the glutamate–glutamine cycle, and balanced glutamate/GABAergic transmission. AMPA, α-amino-3-hydroxy-5-methyl-4-isoxazolepropionic acid receptor; BDNF, brain-derived neurotrophic factor; CB, cannabinoid receptor; EAATs, excitatory amino acid transporters; eCBs, endocannabinoids; ER, endoplasmic reticulum; GDNF, glial cell line-derived neurotrophic factor; Gln, glutamine; Glu, glutamic acid; IFNγ, interferon gamma; IL, interleukin; mGluRs, metabotropic glutamate receptors; NGF, nerve growth factor; NMDA, N-methyl-D-aspartate receptor; NO, nitrogen monoxide; PGD_2_, prostaglandin D_2_; QUIN, quinolinic acid; ROS, reactive oxygen species; TGFβ, transforming growth factor-beta; TNFα, tumor necrosis factor alpha; VGCC, voltage-gated calcium channels.

One example of active involvement of the ECS in mediating cellular communication is the functional coupling of microglia and synapses during normal synaptic activity as well as excitotoxic injury. As previously suggested, microglia are a crucial source of de novo produced AEA and 2-AG under basal conditions and during neuroinflammation [75,76,77]; however, high glutamate application induces a prominent 2-AG overproduction in neurons [76,78]. Under these conditions the neuronal production of AEA increases only slightly, while the production of two putative endocannabinoids, homo-gamma-linolenylethanolamide and docosatetraenylethanolamide remains unchanged [76]. LPS-induced systemic inflammation in mice is accompanied by the increase in basal glutamate levels in the prefrontal cortex [47]. Elevated glutamate may originate from both inflammation-associated decrease in uptake, and neuronal and non-neuronal (from astrocytes and microglia) glutamate release. Glutamate flow favors the spatial cooperation between dendritic spines and ramified microglial cells and induces microglial process extension toward neurons (Figure 4). 2-AG induces chemokinesis (random motion increased by a chemical stimulus) and chemotaxis, (directed cell migration along a chemical gradient) in microglia cells [76,79]. In line with this, activated microglia express CB_2_ receptors at the leading edge of their motile protrusions [76]. Arachidonylcyclopropylamide (ACPA)-induced migration of BV-2 microglia could be blocked by the highly selective CB_2_ antagonist SR145528 [80]. Similarly, the migratory responses towards 2-AG and the synthetic cannabinoid CP 55,940 were inhibited by CB_2_ receptor antagonism [76,79]. We hypothesize that eCBs released at sites of synaptic activity (or injury) may act as a chemoattractants to recruit microglia in a CB_2_-dependent manner, toward neuroinflammatory lesion sites. Moreover, in organotypic hippocampal slice cultures, 2-AG mediated neuroprotection against NMDA-induced excitotoxicity by acting explicitly on abnormal-cannabidiol (abn-CBD)-sensitive receptor, putative GPR18, on microglial cells [81]. 

There is an activity-dependent modification of microglia–synapse contacts in vivo. Ischemic brain is characterized by the markedly prolonged contact time between microglial processes and synaptic structures and wrapping of microglial processes around the synapse, followed by the disappearance of presynaptic boutons [82].

One of the mechanisms by which microglia eliminate presynaptic boutons and axons is trogocytosis [83], a process described in the immune system as a non-apoptotic mechanism for the capture of membrane components that differs from phagocytosis and involves the engulfment and clearance of cellular structures larger than 1 µm [84]. In a mouse model of cortical multiple sclerosis in vivo imaging demonstrated that cortical inflammation disrupts circuit activity, which coincides with a widespread, but reversible, loss of dendritic spines. Under these circumstances, spines displaying local calcium accumulations are eliminated by invading macrophages or resident activated microglia [85]. 

Acute microglia activation is accompanied by the release of glutamate, quinolinic acid, proinflammatory cytokines (IL-1β, TNF-α, IL-2, IL-6), chemokines—macrophage inflammatory protein-1α (MIP-1α) and monocyte chemoattractant protein-1 (MCP-1), and free arachidonic acid. Quinolinic acid produced exclusively in activated microglia and macrophages, is a NMDA receptor agonist and mediates excitotoxicity during immune response. By contributing to destabilization of the cytoskeleton in astrocytes and endothelial cells, quinolinic acid decreases the integrity of the neurovascular unit and increases the influx of BBB impermeable quinolinic acid from the periphery [86]. Produced excitotoxic molecules and proinflammatory cytokines intensify free radical generation and lipid peroxidation, which provoke mitochondrial dysfunction and further exacerbate the excitotoxicity.

Microglia largely define the fate of damaged synaptic contacts and cells and promote the resolution of neuroinflammation and regeneration by releasing brain-derived neurotrophic factor (BDNF) and cytokines with dual (pro- and anti-inflammatory) potential, like TGF-β and IL-10.

## 5. The Role of Retrograde Endocannabinoid Signaling in the Tuning of Synaptic Strength

### 5.1. Synaptic Plasticity in Glutamatergic Synapses

When glutamate levels reach a certain concentration in the synaptic cleft, it binds to AMPA receptors and induces Na^+^ influx, which is registered as excitatory postsynaptic potentials (EPSP) of certain amplitudes. Due to the presence of the GluR2 subunit the majority of AMPA receptors in the CNS are impermeable to Ca^2+^ [87] and postsynaptic Ca^2+^ influx triggered by glutamate is mainly mediated by NMDA receptors. 

**Figure 5:**
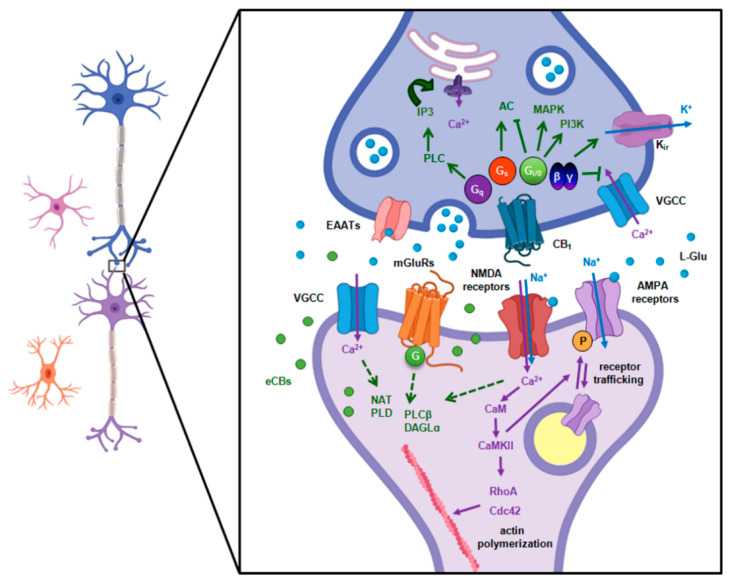
The stimuli involved in the establishment of synaptic plasticity and retrograde eCB signaling in the glutamatergic synapse. Glutamate release from the presynaptic terminal activates ionotropic (NMDA, AMPA, and kainate) and metabotropic glutamate receptors (mGluRs), that together with the Ca^2+^ influx through VGCC are potent triggers for eCB production (indicated in green). Ca^2+^ influx trough NMDA receptors is involved in the regulation of AMPA receptor trafficking, spine enlargement, and plastic changes in the strength of the synaptic transmission (indicated in purple). AC, adenylate cyclase; AMPA, α-amino-3-hydroxy-5-methyl-4-isoxazolepropionic acid receptor; CaM, calmodulin; CaMKII, calcium-calmodulin dependent protein kinase II; Cdc42, cell division control protein 42 homolog; DAGL, diacylglycerol lipase; EAATs, *e*xcitatory amino acid transporters; eCBs, endocannabinoids; Glu, glutamic acid; IP3, inositol 3-phosphate; K_ir_, inward-rectifier potassium channel; MAPK, mitogen-activated protein kinase; mGluRs, metabotropic glutamate receptors; NAT, N-acyltransferase; NMDA, N-methyl-D-aspartate receptor; PI3K, phosphatidylinositol 3-kinase; PL, phospholipase; RhoA, Ras homolog family member A; VGCC, voltage-gated calcium channels.

Influx of Ca^2+^ through NMDA receptor channels activates a range of kinases, primarily Ca^2+^/calmodulin-dependent protein kinase II (CaMKII) [88,89], which in turn activates Rho GTPases, Cdc42, and RhoA [90]. This reorganizes the postsynaptic density via * remodeling of the actin cytoskeleton and transient (~5 min) enlargement of the spine (Figure 5); ** enhanced trafficking of AMPA receptors to post-synaptic sites as a result of their redistribution from recycling endosome to the plasma membrane [91,92], and *** increased single-channel conductance of AMPA receptors as a result of direct phosphorylation [93].

Synaptic recruitment of Ca^2+^-permeable AMPA receptors via CaMKI is also suggested to contribute to signaling pathways that drive the spine enlargement via actin polymerization [94]. Thus, following the repeated cycles of activation, the amplitude of evoked EPSC increases, i.e., is potentiated (long-term potentiation, LTP). This effect is typical for excitatory neurotransmission and persists in synapses depending on the stimulus mode. 

In contrast, long-term depression (LTD) is a long-lasting drop in the efficiency of synaptic transmission as a result of a decrease in postsynaptic receptor density and/or presynaptic neurotransmitter release. While for development of LTP the activation of certain protein kinases is essential, LTD induction is dependent on protein phosphatase activity and target dephosphorylation. Prolonged 1 Hz stimulation leads to Ca^2+^ rise and calmodulin-dependent activation of calcineurin (protein phosphatase 2B, PP2B) [95], which via serine/threonine protein phosphatases PP1 or PP2A, results in the dephosphorylation of AMPA receptors [96], decrease of their channel conductance, and arrest of their recycling [97]. Dephosphorylation of the transcription factor cAMP response element binding protein (CREB) in the hippocampal area CA1 in vivo is suggested to be one of the mechanisms through which these protein phosphatases contribute to the prolonged maintenance of LTD [98].

The development of LTP or LTD depends on whether the frequency of the stimulation is higher than threshold frequency [99], with the postsynaptic rise of Ca^2+^ as a main determinant of the development of LTD or LTP. LTD and LTP are well-characterized molecular mechanisms underlying learning and memory formation and are induced in experiments with high- or low-frequency stimulations (repetition of hundreds of pre- or postsynaptic spikes). In the striatum of rodents, spike-timing-dependent potentiation (STDP), a phenomenon describing the dependence of strength of synaptic transmission on the timing between the neuron’s output and input action potentials (spikes), is observed for 75–100 pairings, disappears for 25–50 pairings and re-emerges for 5–10 pairings. STDP that is induced by very few pairings is independent from NMDA receptors but mediated by 2-AG and AEA, acting on both CB_1_ and TRPV1 [100]. 

### 5.2. Endocannabinoid-Mediated Synaptic Plasticity

Stimulation of postsynaptic neurotransmitter receptors and sustained Ca^2+^ influx is the potent trigger for the production of eCBs and their congeners [78,101] in a neuronal activity-dependent manner. The released neurotransmitter activates ionotropic neurotransmitter receptors, G_q_-coupled metabotropic receptors (group I of metabotropic glutamate receptors, mGluRs, dopamine receptors D2, M1, and M3 muscarinic acetylcholine receptors, M1/M3 mAChRs), and/or voltage-gated calcium channels. This induces a burst of Ca^2+^ in the postsynapse and the production of eCBs, primarily AEA and 2-AG, which via CB_1_ and CB_2_ receptors modulate the presynaptic release of neurotransmitters. Thus, generation of AEA and 2-AG in the brain shows spatial variations and depends on complement of neurotransmitter receptors on certain neurons. Endocannabinoid-mediated synaptic potentiation can be realized in neurons receiving/sending inputs via taken activated synapses (homosynaptic) or in other neurons that do not directly contact taken activated synapses (heterosynaptic). 

*Endocannabinoid-mediated long-term depression (eCB-LTD).* CB_1_ and CB_2_ are G_i/0_ protein-coupled receptors that, upon ligand binding, inhibit adenylate cyclase activity and cAMP production, negatively regulate voltage-gated calcium channels (VGCC), and activate inwardly rectifying potassium (K_ir_) channels and MAP kinase. This decreases the Ca^2+^ influx into the presynaptic terminal, lowers the probability of Ca^2+^-dependent fusion of synaptic vesicles and attenuates the presynaptic neurotransmitter release. Synaptic vesicle recycling is a highly dynamic multistep process mediated by SNARE-proteins and other regulatory proteins, with Ca^2+^ influx being a key factor for docked and primed synaptic vesicles to enter the fusion step [102]. Together with the activity-dependent production of eCBs and activation of CB_1_, presynaptic activity is essential for this type of plasticity as it determines the afferents in which eCB-LTD will be induced [103]. Presynaptic activity dependence is mediated by presynaptic NMDA autoreceptors that detect the release of glutamate. Thus, timing-dependent-LTD is induced only under coincident activation of presynaptic NMDA and CB_1_ receptors [104]. Unlike LTP, which lasts from minutes to hours, short-term plasticity (STP) is maintained for tens of milliseconds to a few minutes at a maximum.

*Depolarization-induced suppression of inhibition (DSI)/depolarization-induced suppression of excitation (DSE).* DSI/DSE is a form of STP realized in the inhibitory (GABAergic) and excitatory (glutamatergic) synapses, respectively, and is induced by depolarization of the postsynaptic terminal and Ca^2+^ influx through VGCC. Pharmacological experiments favor a role for 2-AG rather than AEA or noladin ether (2-arachidonyl glyceryl ether) as the relevant endocannabinoid to elicit DSE [105]. Glutamate spillover may profoundly affect network excitability by shifting the duration of eCB-mediated inhibition at GABA synapses. Metabotropic glutamate receptors are involved in the control of the duration of DSI, most likely through heterologous desensitization of CB_1_ [106]. Thus, glutamate-mediated excitotoxicity can significantly modify establishment of various forms of synaptic plasticity and balance in glutamate-/GABAergic signaling [47].

*Synaptically-evoked (or metabotropic-induced) suppression of inhibition/excitation (SSE/SSI).* SSE/SSI is a form of STP, driven by activation of postsynaptic metabotropic (G_q/11_-coupled) neurotransmitter receptors, subsequent activation of phospholipase C (PLC) and DAGL with generation of 2-AG and suppression of neurotransmitter release via presynaptic CB_1_ receptors [107]. If the synaptic stimulation is profound, produced eCBs can reach more distant sites and mediate the plasticity heterosynaptically.

*TRPV1-mediated synaptic plasticity.* TRPV1-mediated synaptic plasticity demonstrates the interplay between the eCB and endovanilloid system. This type of synaptic plasticity is mediated by binding of AEA to TRPV1 at the postsynapse and results in Ca^2+^-calcineurin and clathrin-dependent internalization of AMPA receptors, which provoke LTD of excitatory transmission [108,109]. TRPV1 integrates sensation of physical and chemical stimuli and is activated by temperature greater than 43 °C, acidic conditions, vanilloids like capsaicin, or endocannabinoids such as AEA, N-arachidonoyl dopamine, and N-oleoyl dopamine. TRPV1 is well known for its role in transmission of neuropathic (inflammatory) pain. At the same time TRPV1 mediates LTD in the hippocampus and 12-(S)-hydroperoxyeicosatetraenoic acid (12-(S)-HPETE), an endogenous eicosanoid released during synaptic stimulation, acts at TRPV1 receptors to trigger LTD [110].

It is hypothesized that eCB-mediated LTP is induced only when massive Ca^2+^ rise is observed and high levels of 2-AG are produced (i.e., following simultaneous activation of several postsynaptic neurotransmitter receptors, TRPV1, VGCC) [100]. The observations that2-AG and AEA mediate different forms of plasticity with the involvement of CB_1_, TRPV1, and mGluRs receptors depending on the brain region, characterize the eCB system as a polymodal signal integrator that allows the diversification of synaptic plasticity in a single neuron [111].

The interference of NAEs and other eCB congeners with enzymatic degradation or endocannabinoid signaling suggests their role in tuning the activity of primary eCBs. NAEs, monoacylglycerols, and certain N-acylneurotransmitters compete with AEA or 2-AG for FAAH-mediated degradation, thus extending their lifetimes [112] and their ability to interact with cannabinoid receptors. This “entourage effect” of the non-cannabinoid 2-acylglycerols and NAEs may serve as an additional fine regulator of cannabinoid activity. The overall interplay and metabolism of endogenous ligands of CB_1/2_, TRPV1, GPR55, and GPR18 is now integrated in the “endocannabinoidome” and demonstrates that this system is an essential player not only in many aspects of behavior, cognition, and memory, but also mediates inherent protective mechanisms of the neuro-immune interface. 

## 6. Neuroinflammation-Induced Synaptopathy and Neurodegenerative Diseases

Neuroinflammation is a common feature of acute and chronic neurodegenerative disorders such as Alzheimer’s and Parkinson’s disease, viral infections of the CNS, stroke, paraneoplastic disorders, traumatic brain injury, and multiple sclerosis. Neuroinflammation is typically characterized by activation of immunocompetent glia cells (microglia and astroglia), release of cytokines, prostaglandins, and reactive oxygen species, the impairment of the blood–brain-barrier (BBB) integrity and resultant infiltration of peripheral immune cells. 

### 6.1. Microglia

Microglia are residential innate immune cells that perform primary immune surveillance and macrophage-like activities of the CNS. In a non-stimulated state, microglia contribute to CNS development and maintain tissue homeostasis by supporting neuronal survival, cell death, and synaptogenesis [113]. However, microglial cells can be activated by various pathological stimuli during infections, brain trauma, stroke, and neurodegeneration [114]. Activated microglia are characterized by increased proliferation and the production and secretion of a wide spectrum of immune mediators such as cytokines, chemokines, prostaglandins, and reactive oxygen intermediates [115,116,117,118]. The production of cytokines and chemokines can facilitate the recruitment of peripheral leukocytes into the brain [119]. During neuroinflammation, activated microglia migrate to the site of injury or infection and perform pivotal immunological functions such as phagocytosis of invading microorganisms and removal of dead or damaged cells [120,121]. However, chronic activation of microglia is generally considered to be detrimental for neuronal health [122].

In response to activation, microglia can polarize to either a pro-inflammatory M1 phenotype or an anti-inflammatory M2 phenotype, although microglial activation states have been recognized to be more complex [123]. Various stimuli induce the classical M1 activation state of microglia such as LPS, interferon (IFN)-γ, amyloid β (Aβ), and α-synuclein [118,124,125,126]. Toll-like receptor 4 (TLR4), a member of the pattern recognition receptor family, mediates innate immunity and is abundantly expressed in microglia [127]. In fact, TLR4-dependent microglial activation has been observed in various neurodegenerative diseases like Alzheimer’s disease (AD) and Parkinson disease (PD) [128,129]. In addition, TLR4 is also responsible for chronic neuroinflammation after stroke and spinal cord injury leading to brain damage [130]. TLR4 can be activated by multiple pathogen-associated molecular patterns (PAMPs), such as LPS which is a major component of the outer membrane of Gram-negative bacteria [130]. LPS is one of the most extensively studied TLR4 ligands to understand the mechanism of microglial activation in neurodegeneration [127]. In addition to the above-mentioned pro-inflammatory response, microglia can also adopt an anti-inflammatory M2 phenotype. After M1 microglia attack invading organisms to limit tissue damage, anti-inflammatory M2 microglia are involved in phagocytosis of cellular debris and wound healing [123]. Another more immuno-suppressive phenotype is induced by the cytokines IL-10 and TGF-β, or by apoptotic cells [123]. 

Microglia are a crucial source of AEA and 2-AG under basal conditions and during neuroinflammation [75,76,77]. When stimulated with ATP (released from damaged tissue) microglia produce 2-AG themselves [77]. CB_2_ receptor expression is upregulated in microglia stimulated with pro-inflammatory cytokines [131], indicating a significant role of CB_2_ in the regulation of neuroinflammatory states. Further, it was demonstrated that 2-AG and PEA affect microglial cells and lead to a decrease in the number of damaged neurons after excitotoxical lesion in organotypic hippocampal slice cultures. 2-AG activated the abnormal cannabidiol (abn-CBD) receptor and PEA was shown to mediate neuroprotection via PPAR-α [132]. The CB_2_ agonist AM1241 has also been shown to attenuate microglia activation by reducing the expression of the inducible nitric oxide synthase and shifting their phenotype from M1 to M2 [133].

Microglia constantly scan their environment and due to the long processes directed towards synapses can monitor and respond to the functional status of synapses. Microglia contribute to neuronal circuit maturation and are involved in both synapse induction and elimination. During neuroinflammation, prolonged activation of microglial cells attracted to lesion sites can exacerbate neuronal damage. It is anticipated that 2-AG, produced by overstimulated neurons, induces microglial migration [76] and proliferation [134]. 

### 6.2. Astrocytes

Astrocytes are the largest and most abundant group of glial cells in the CNS and play a vital role in regulating CNS homeostasis, synaptic transmission and plasticity, and neuroprotective effects. Glia respond to neuronal activity with the elevation of their internal Ca^2+^ concentration, which triggers the release of mediators of glial origin. The term ‘tripartite synapse’ describes the bidirectional communication between astrocytes and neurons, where nearby astrocytes respond to synaptic activity and, vice versa, regulate synaptic transmission and plasticity [135]. Perisynaptic Schwann cells and synaptically associated astrocytes are viewed as integral modulatory elements of tripartite synapses. 

CB_1_ activation in astrocytes amplifies Ca^2+^ influx and promotes the release of gliotransmitters, like glutamate (gliotransmission), which modulate the target response at pre- and postsynaptic sites [24]. Impairment of spatial working memory and in vivo long-term depression (LTD) of synaptic strength at hippocampal CA3-CA1 synapses, induced by an acute exposure of exogenous cannabinoids, is due to the activation of astroglial CB_1_ and is associated with astroglia-dependent hippocampal LTD [136]. 

Due to their proximity to blood vessels and other resident cells within the CNS such as neurons, microglia, and oligodendrocytes, astrocytes play a crucial role in BBB maintenance and permeability. In addition, astrocytes are involved in modulating the innate immune response by regulating inflammatory factors, such as cytokines, chemokines, complement fragments, reactive oxygen, or reactive nitrogen species. However, dysfunctional astrocytes seem to play an important role in the onset of neurodegenerative diseases such as AD and ALS (reviewed in [137]). 

Astrocytes become activated or reactivated during various pathological conditions such as stroke, trauma, tumor growth, and neurodegenerative diseases. Following the M1/M2 phenotype classification of microglia and macrophages, neuroinflammation can induce two types of reactive astrocytes, termed A1 and A2 [138]. In A1 reactive astrocytes the pro-inflammatory NF-κB pathway is upregulated leading to the release of complement factors [139] that are destructive to synapses and to the secretion of neurotoxins and pro- and anti-inflammatory mediators such as PGD_2_, IFN-γ, TNF-α, IL-1-β, and TGF-β, respectively [140,141,142]. In A2 reactive astrocytes enhanced STAT3 activity has been observed [143]. Moreover, A2 reactive astrocytes can upregulate many neurotrophic factors such as thrombospondins [144] and brain-derived neurotrophic factor (BDNF) [145,146], which promote either survival and growth of neurons or synaptic repair. 

Accumulating evidence supports the role of astrocytes as a source of eCBs. It has been shown that astrocytes have the potential to produce 2-AG in response to ATP [147], endothelin [148], and CB_1_ receptor activation [75,149]. Furthermore, the secretion of AEA, homo-gamma-linolenylethanolamide (HEA), and docosatetraenoylethanolamide (DEA) by activated mouse astrocytes has been confirmed [75]. 

Of note, not only microglial cannabinoid receptors but also astroglial CB_1_ and CB_2_ receptors play critical roles in the response to neuroinflammation [33]. Activation of astroglial CB_1_ receptors protects against ceramide-induced oxidative stress and apoptosis [150,151] and activation of both CB_1_ and CB_2_ receptors seems to prevent LPS-induced nitric oxide (NO) release by cultured astrocytes [152]. Accordingly, 2-AG has been shown to maintain glutamine synthase expression in astrocytes in a MAPK-dependent manner and thus to protect astrocytes from LPS exposure [153]. 2-AG also seems to reduce the astrocytic production of chondroitin sulfate proteoglycan, that accumulates in MS lesions and is thought to be linked to the failure to regenerate, impeding oligodendrocyte precursor cell differentiation, and neuronal growth [154]. Moreover, 2-AG protects astrocytes exposed to oxygen-glucose deprivation through a blockade of NDRG2 signaling and STAT3 phosphorylation [155]. 2-AG and PEA have been shown to attenuate amyloid β-induced astrocyte activation and PEA increased 2-AG production in astrocytes [156,157,158]. PEA is also suggested to improve neuronal survival by possibly counteracting reactive astrogliosis [159,160]. PEA- and OEA-mediated inhibition of astrocyte activation seems to involve PPAR-α [161,162]. Furthermore, AEA has been shown to elicit glutamate release through astrocytic CB_1_ receptor activation in the core of nucleus accumbens in rats [163]. 

Astrocytes isolated from mice with acute experimental autoimmune encephalomyelitis (EAE) exhibited reductions in all endocannabinoid metabolism-associated genes, with the exception of *Faah*, which persisted in chronic disease and was associated with reduced *Cnr1* transcript levels both at acute and recovery phases [164]. Astrocytic- together with neuronal MAGL seems to be responsible for converting 2-AG to prostaglandins and thus protects the nervous system from excessive CB_1_ receptor activation [165].

### 6.3. Cytokines Involved in Neuroinflammation

Besides microglia and astrocytes, endothelial cells and other glial cells, may produce cytokines and chemokines. Common cytokines which are produced in response to brain injury or during neurodegenerative diseases able to induce neuronal cytotoxicity are IL-6, IL-1β, and TNF-α [166]. Moreover, sustained release of these cytokines leads to a compromised BBB [167]. Subsequently, peripheral immune cells such as macrophages, neutrophils, monocytes, T cells, and B cells are able to migrate into the brain. This process exacerbates and contributes to chronic neuroinflammation and neurodegeneration. For instance, following traumatic brain injury, IL-1β induces neuronal apoptosis, BBB breakdown, recruitment of immune cells, as well as the production of pro-inflammatory mediators [168]. Moreover, during spinal cord injury, the secretion of pro-inflammatory mediators including IL-1β, inducible nitric oxide synthase (iNOS), IFN-γ, IL-6, IL-23, and TNF-α is followed by the activation of local microglia and attraction of various immune cells such as naive bone-marrow derived macrophages [169]. Upon infiltration of the injured site, macrophages undergo phenotype switching from M2 phenotype to M1-like phenotype. Noteworthy, normal aging is often associated with an increased number of activated microglia in the brain which are involved in altered synaptic plasticity mechanisms in the hippocampus, including LTP and thereby reduce memory performance [170]. Moreover, aged brains show homeostatic imbalance between anti-inflammatory and pro-inflammatory cytokines increasing the risk for neurodegenerative diseases such as AD. Although the pro-inflammatory cytokines may cause cell death and tissue damage, they are also involved in tissue repair [171]. For example, TNF-α causes neurotoxicity at early stage, but contributes to tissue growth at later stages of neuroinflammation. 

Several studies have highlighted the regulatory effects of the ECS on neuroinflammatory conditions by modulating the production of cytokines. For instance, 2-AG was shown to prevent the overexpression of TNF-α, IL-1β, and iNOS in a murine model of SO_2_-induced brain inflammation [172]. MAGL-deficiency leading to increased 2-AG levels also reduced brain PGE_2_ and pro-inflammatory cytokine levels following peripheral LPS administration in mice [173]. Selective pharmacologic inhibition of ABHD6 diminished cytokine and chemokine production in a murine model of neuropathic pain [174], and the MAGL inhibitor CPD-4645 significantly reduced IL-1β and IL-6 brain levels after systemic LPS challenge [175]. The FAAH inhibitor URB597 attenuated increased TNF-α and IL-1β levels in the hippocampi of aged mice [176] and decreased Iba-1, TNF-α, IL-6, and monocyte chemoattractant protein-1 (MCP-1) levels in the hippocampus of ethanol-exposed rats [177]. The FAAH inhibitor PF3845 increased levels of AEA, OEA, and PEA in the frontal cortex and hippocampus of rats [178]. Furthermore, this increase in FAAH substrate levels was associated with a robust attenuation in TNF-α, IL-6, and IL-1β levels in the prefrontal cortex. PEA has been shown to reduce pro-inflammatory cytokines after traumatic spinal cord [179] and brain injury [180,181], in a model of sciatic nerve crush [182], in Parkinson’s disease models [183,184] and in MS patients [185]. Further, OEA administration significantly reduced plasma and brain TNF-α levels after LPS application [186] and SEA was recently shown to suppress increased TNF-α and TGF-β1 levels in the prefrontal cortex of LPS challenged mice [47].

CB_2_ receptor agonism has been shown to decrease brain levels of pro-inflammatory cytokines induced by LPS application [187], intracerebral hemorrhages [188], or surgery [189] as well as in a model of PD [190]. At the same time the CB_1_ receptor inverse agonist SR141716A (rimonabant) and the CB_2_ receptor antagonist SR144528 significantly reduced LPS-induced IL-1β production in the brain [191] whereas SR141716A was also shown to increase pro-inflammatory cytokines in an EAE model [192]. Neuroprotective effect of SR141716A was shown in the retinal degeneration model [193] and in permanent photothrombotic cerebral ischemia [194]. These findings indicate that manipulation of CB_1_ or CB_2_ receptors may have therapeutic value in neuroinflammation; however, due to the complexity of the ECS, this concept remains to be carefully considered.

### 6.4. Effects of Endocannabinoids and Related Compounds on Neurodegenerative Diseases

Not only inflammatory disorders or tissue injury, but also neurodegenerative diseases are accompanied or caused by neuroinflammation. Neurodegeneration also refers to chronic and progressive loss of neuronal functions in the brain and spinal cord. Particularly, AD is characterized by amyloid β plaques and neurofibrillary tangles that cause a decline in memory and cognitive abilities. In addition to neuroinflammation, systemic infection and inflammation, characterized by a substantial amount of proinflammatory mediators in the circulation, have also been correlated to increased risks of developing AD [195]. Chronic inflammation is also a hallmark of PD, which is characterized by the loss of dopaminergic neurons and the presence of α-synuclein-containing aggregates in the substantia nigra pars compacta [196]. Huntington’s disease (HD) is a devastating neurodegenerative genetic disorder associated with progressive loss of a specific type of neurons found in the striatum and cortex [197]. Unfortunately, the relationship between neuroinflammation markers and the disease pathology is still poorly understood [198]. Amyotrophic lateral sclerosis (ALS) is indicated by the degeneration of motor neurons [199] and characterized by the occurrence of a neuroinflammatory reaction consisting of activated glial cells, mainly microglia and astrocytes, and T cells [200]. Of the neurodegenerative diseases, multiple sclerosis (MS) is an exception because neuronal death in MS is considered to be secondary to the initiating activity of autoreactive T cells that target myelin [201]. While neuroinflammation in MS involves infiltration of peripheral immune cells, breakdown of the BBB, and activation of CNS-resident glial cells, neuroinflammation in the other neurodegenerative diseases is more restricted to glial cell activation and inflammatory reactions in the parenchyma [201].

Several animal models and human studies have demonstrated that the ECS significantly influences the development of neuroinflammation and the progression of brain injury and neurodegenerative diseases [1,2,3]. Using a model of cerebral focal ischemia, it was shown that exogenously administered AEA and 2-AG in combination reduced infarct size in rats, but with no facilitatory effects beyond AEA or 2-AG alone [202]. Other studies reported neuroprotective effects of exogenous AEA [203] and 2-AG [56] under traumatic brain injury (TBI). Several studies documented the positive effects of PEA in experimental and clinical studies of TBI, spinal cord injuries, pain, cerebral ischemia, PD, and AD [179,180,181,183,184,185,204,205,206,207,208]. Moreover, the stimulation of CB_1_ and CB_2_ has been shown to be beneficial in neurodegenerative disorders such as AD and PD (reviewed in [209,210]). In contrast, the CB_1_ inverse agonist SR141716A (rimonabant) showed promising results in PD preclinical studies [211,212]; however, clinical studies with SR141716A failed to improve motor disability in PD patients [213]. Importantly, SR141716A, which has also been approved as an effective anti-obesity drug, shows a serious psychiatric side effect profile, and thus has been withdrawn from the pharmaceutical market worldwide. These contradictory data, indicate the interference of CB_1_ inverse agonists/antagonists with a basal ECS tone, prevailing in healthy conditions and essentially involved in the homeostatic regulation of brain function.

It has been observed that CB_2_ receptors and FAAH are selectively overexpressed in neuritic plaque-associated glia in AD [214], especially in reactive astrocytes and activated microglial cells [215]. In this regard, the use of hydrolase inhibitors has been mentioned as a promising therapeutic option for neuroinflammatory and neurodegenerative diseases. Treatment with MAGL and FAAH inhibitors, which have the capacity to increase the level of eCBs indirectly, leads to anxiolytic, antidepressant, and anti-inflammatory effects, and reduces amyloid β deposition and inhibition of the death of dopaminergic neurons, which are associated with the pathogenesis of AD and PD, respectively (reviewed in [216,217]). An overview of in vivo studies reviewing the effects of MAGL and FAAH inhibitors in neuroinflammation and neurodegenerative diseases, is given in Table 3. Several FAAH and MAGL inhibitors entered clinical phase I and II studies for neurological disorders such as pain, anxiety, Tourette syndrome, and cannabis withdrawal. Moreover, dual FAAH/cholinesterase inhibitors which might be beneficial for neurodegenerative diseases are currently under development [218]. In addition, an increasing number of studies have characterized the beneficial effects of NAAA inhibitors, which prevent the degradation of NAEs, in preclinical studies of pain and (neuro-) inflammation [219,220,221] (Table 3). However, in a phase I study, the FAAH inhibitor BIA 10-2474 resulted in severe adverse events such as lethal toxic cerebral syndrome [222], leading to the discontinuation of several clinical studies employing FAAH inhibitors. Thus, there is an urgent need of further research to develop highly specific, short-acting, indirect cannabinoid therapies with better safety profiles.

## 7. The Role of the Blood–Brain Barrier Integrity in Restriction of Systemic Inflammation

CNS dysfunction associated with systemic infection is common and includes symptoms such as sickness behavior and delirium. In the context of sepsis, CNS dysfunction is known as septic encephalopathy. A key step in the pathogenesis is the systemic production of pro-inflammatory cytokines such as TNF-α and IL-1β, which then act on the brain. Cytokine transport systems of the BBB are likely to play a role in permitting the passage of these signals [241]. Moreover, AD, MS, and CNS dysfunction in systemic infection are examples of conditions which are primarily neurodegenerative, neuroinflammatory, or systemic. In many cases it is not clear whether BBB changes are the cause or consequence of neuropathology, and it is possible that BBB changes and neuropathology drive each other in a self-perpetuating manner, contributing to disease progression.

### 7.1. Structure of the Blood–Brain Barrier

Histologically the BBB is a specialized multi-layered unit composed of a thick continuous glycocalyx, non-fenestrated endothelial cells with reduced vesicular activity and linked by tight junctions, two basement membranes (vascular basement membrane and glia limitans), and astrocytic end-feet. All elements of this ‘neurovascular unit’ contribute to the functional BBB. At the molecular level, there are ectoenzymes, receptors and transporters which regulate or reverse traffic across the BBB. Together, these components enable a stable CNS environment to minimize traffic of inflammatory cells and molecules, local inflammation, and prevent potential neuronal damage.

### 7.2. The BBB in Systemic Inflammation

Systemic inflammation, as induced by LPS, can lead to disruptive and non-disruptive BBB changes. Disruptive BBB change is accompanied by endothelial cell damage or tight junctional modifications, while non-disruptive change occurs at a molecular level. Identified mechanisms of LPS-induced disruptive BBB change include modification of tight junctions, endothelial damage and apoptosis, degradation of glycocalyx, breakdown of glia limitans, and astrocyte alteration (reviewed in [242]). Moreover, several reports demonstrate that systemic inflammation upregulates several endothelial cell receptors and transporters, induces cytokine production by endothelial cells, modulates astrocyte function, and enhances pathogen neuroinvasion without any visible changes in the BBB architecture [242].

### 7.3. The Role of the ECS in the Maintenance of the Blood Brain Barrier

2-AG is the most abundant endocannabinoid in the CNS and is elevated after brain injury and during neuroinflammation. Because of its rapid hydrolysis, however, the compensatory and neuroprotective effect of 2-AG is short-term. It has been shown previously that 2-AG decreases BBB permeability and inhibits the acute expression of the main proinflammatory cytokines TNF-α, IL-1β, and IL-6 [243]. Moreover, several reports demonstrated that inhibition of 2-AG and AEA degradation supports the BBB integrity in experimental traumatic brain injury [244,245] and ischemic insults [175]. Accordingly, CB_2_ agonists prevents BBB damage in several experimental models of brain injury and neurodegenerative disease [188,246,247,248,249,250,251,252,253,254,255,256,257]. Previously, Hind and colleagues suggested that AEA, OEA, and PEA may play an important modulatory role in normal BBB physiology, and afford protection to the BBB during ischemic stroke [258]. In addition, it has been shown that PPAR-α is involved in the protective effects of OEA and OEA analogues against ischemic brain injury, particularly in terms of BBB disruption [259,260]. A study from Mestre and colleagues suggested that CB_1_ receptor dependent inhibition of vascular cell adhesion molecule (VCAM) 1 is a novel mechanism for AEA-induced leukocyte transmigration trough the BBB [261]. Moreover, CB_1_ receptor blockade reduced leukocyte adhesion to intestinal microvasculature in a mouse model of systemic sepsis [213], potentially also affecting immune cell transmigration at the BBB. The major endogenously produced NAE and one of the most stable among these compounds, SEA prevents leukocyte, especially neutrophil, infiltration into the brain in a murine model of LPS-induced systemic inflammation [47].

### 7.4. Leukocyte Recruitment

Leukocytes entering the brain from peripheral circulation must pass through the BBB, the choroid plexus that forms the blood–cerebrospinal fluid barrier, and through post-capillary venules at the pial surface into subarachnoid and Virchow-Robin perivascular spaces [262,263]. These routes typically operate together and both the paracellular (junctional) and transcellular (non-junctional) mechanisms can be involved in leukocyte trafficking across the neurovascular unit [264]. Although cellular influx into the CNS is physiologically non-disruptive, it may result in disruptive BBB change. Leukocyte recruitment across the BBB in response to systemic inflammation has been demonstrated for lymphocytes [265], neutrophils [266], and monocytes [267]. Mechanistically, systemic inflammation can promote leukocyte transmigration at various points during the two-step passage through the endothelium and glia limitans.

On circulatory immune cells, CB_1_ and CB_2_ receptors are expressed at low levels in healthy human donors. Highest expression levels of CB_2_ were observed on NK-cell, B-cells and monocytes, while low levels could be found in T cells and neutrophils isolated from peripheral blood of healthy donors [268]. Stimulation with pro-inflammatory cytokines, such as IL-6 and TNF-α, enhanced transcription of both CB receptors, while the effect was more pronounced for CB_2_ [269]. In line with this, pro-inflammatory cytokine stimulation of murine bone marrow-derived macrophages increased CB_2_ expression [270], rendering inflammatory macrophages more susceptible to CB signaling. A detailed overview of the reported effects of CB_1_ and CB_2_ receptor activation in leukocytes is given in Table 4. 

## 8. Conclusions

Neuroinflammation is caused and/or accompanied by the infiltration of immune cells through the BBB and secretion of a range of pro-inflammatory cytokines and other molecules with neurotoxic potential. These changes together with glutamate receptor-mediated neurotoxicity and neurodegenerative processes underlie the pathogenesis of several neuropathologies, among them are Alzheimer’s disease, amyotrophic lateral sclerosis, stroke, multiple sclerosis, and Parkinson’s disease. Bacterial and viral infection, traumatic injury, and autoimmune disease compromise the integrity of BBB and favor the transition of systemic inflammation to neuroinflammation. Maintenance or restauration of the selective permeability of the blood–brain barrier is one of the therapeutic strategies under systemic inflammation to prevent systemic inflammation from spreading to the CNS.

The interplay of true eCB and ligands that now belong to expanded ECS allow taking into the consideration all relevant molecular targets for therapy of neuroinflammation-associated neuropathologies. The interference of eCB congeners with enzymatic degradation or endocannabinoid signaling suggests their role in tuning the activity of primary eCBs. The ‘entourage effect’ of the produced non-cannabinoid 2-acylglycerols, NAEs and N-acylneurotransmitters may serve as an additional fine regulator of cannabinoid activity.

Resident microglia and astrocytes are tightly coupled to functions of active synaptic contacts and are highly involved in inflammatory progression, pro-survival changes and resolution of neuroinflammation. These cells promote neuronal survival, synaptogenesis, spine induction, and illumination and protect neurons from toxic metabolites. The contribution of eCB signaling into the functional coupling of neurons, astrocytes, and microglia, suggests that in line with conception of tripartite synapses, microglial cells are equal participants in such communication. Being activated during the immune response, microglial cells contribute to the resolution of neuroinflammation; however, chronic activation of microglia is detrimental to neurons and contributes to the development of synaptopathy in various neurodegenerative diseases. Components of ECS play an active role in the reactivity of these cells during inflammation, attenuate the production of pro-inflammatory cytokines, and mediate neuroprotection against glutamate-receptor mediated excitotoxicity, ischemia, and oxidative stress.

Normal synaptic activity as well as pathological overstimulation of postsynaptic neurotransmitter receptors is a potent trigger for the production of eCBs and non-cannabinoid NAEs. Glutamate-induced endocannabinoids [78] flown from active synapses and injured sites might attract resident microglial cells [76,79]. Tight structural and functional cooperation of synaptic contacts, astrocytes, and microglia enables highly dynamic response to synaptic events.

Retrograde endocannabinoid signaling is implicated in several forms of short- and long-term synaptic plasticity. These lipid mediators reach the presynaptic sites of the same or other synaptic contacts and by binding to CB_1/2_ inhibit the synaptic vesicle fusion and neurotransmitter release. This is how synaptic contacts dynamically tune their own strength and can potentiate/depress the response depending on present inputs.

Released eCBs have a restricted area of action due to short half-lives and differences in CB receptor expression on cells in close vicinity. Thus, the concentration gradient of eCBs is formed on the site of their synthesis. This makes the pharmacological inhibition of eCB degradation primarily effective in injured sites, where they are actively produced. Novel, highly selective inhibitors of FAAH and MAGL with a good safety profile may become prospective agents with anti-nociceptive, anxiolytic, and anti-inflammatory activity and targeted action.

The overall interplay and metabolism of endogenous ligands of CB_1/2_, TRPV1, GPR55, and GPR18 is now integrated in the “endocannabinoidome”, which is actively involved in the intrinsic response to inflammation and neuroinflammation. The polymodality of this system provides a wide field for development of highly efficient neuroprotective agents for the therapy of inflammation-associated synaptopathy.

## Figures and Tables

**Table 1:** Schematic representation of functional contribution of CB_1/2_ in neurons, astrocytes and microglia.

Receptor	Location/ Cell Type	Process Regulated	Intracellular Pathway Involved	Physiological Relevance
CB_1_	Neurons	Presynaptic vesicular neurotransmitter release	Multiple, including inhibition of AC and VGCC; resulting in regulation of [Ca^2+^]_i_	Synaptic plasticity
		[Ca^2+^] influx		Neuronal survival
	Astrocytes	Glutamate–glutamine cycle	Upregulation of glutamine-synthase	Perisynaptic glutamate scavenging, prevention of excitoxicity
		Gliotransmission	Ca^2+^ influx	Modulation of synaptic strength; role in synaptic plasticity
	Endothelial cells	Growth and proliferation	Coupled to the MAP kinase cascade	Maintenance of BBB integrity and selectivity
CB_2_	Ventral tegmental area dopamine neurons	Neuronal excitability	Modulation of K^+^ channels [30]	Long-term neuronal hyperpolarization; synaptic plasticity
	Hippocampal CA3/CA2 pyramidal neurons		Activation of the Na+/Bicarbonate co-transporter [34]	
	Microglia	Migration	Extracellular signal-regulated kinase (ERK) 1/2 signal transduction pathway	Shift between the pro-inflammatory and pro-resolving phenotype

**Table 2:** The profiles and activity of eCBs and their congeners in pathological conditions of the CNS.

Compound	Organism	Disease/Model	Finding	References
2-AG	Mouse	Closed head injury	Increased 2-AG brain levels; neuroprotection	[56]
	Rat	Inflammatory model of pain	Antinociceptive effect	[57]
		Stress-induced analgesia	Increased 2-AG and AEA mid-brain levels; analgesia	[58]
AEA	Mouse	Experimental autoimmune encephalomyelitis	Increased AEA brain levels	[59]
	Human	Multiple sclerosis	Increased AEA levels in cerebrospinal fluid	[59]
	Rat	Focal cerebral ischemia model	Increased AEA brain levels	[60]
NADA	Mouse	Acute systemic inflammation	Reduces inflammation in vivo; increases survival in endotoxemic mice	[61]
2-AG, AEA, PEA	Mouse	Transgenic model of Huntington’s disease	Changed levels of 2-AG, AEA, and PEA in a disease phase- and brain region-specific way	[62]
PEA	Rat	Granulomatous inflammation	Reduced granuloma-induced hyperalgesia	[63]
	Mouse	Paw model of hyperalgesia	Reduced mechanical hyperalgesia	[39]
		Model of neuropathic pain	Anti-allodynic and anti-hyperalgesic effects	[64]
		Alzheimer’s disease	Rescued cognitive deficit and reduced neuroinflammation and oxidative stress	[40]
		Traumatic brain injury model	Reduced edema and brain infractions; blocked infiltration of astrocytes	[65]
SEA	Mouse	LPS-induced neuroinflammation	Decreased activation of resident microglia and leukocyte trafficking into the brain; increased CB_1/2_ expression and 2-AG brain levels	[47]

**Table 3:** FAAH, MAGL, and NAAA inhibitors and their effects in neuroinflammation and neurodegenerative diseases.

Inhibitor	Compound	Disease/Model	Finding	References
FAAH inhibitors	PF-3845	Chronic mouse model of PD	decreased CB_2_ expression	[223]
		TBI mouse model	Enhanced AEA brain levels; reduced neurodegeneration in the dentate gyrus; suppressed production of amyloid precursor protein; suppressed expression of iNOS and COX-2	[224]
	URB597	Mouse model of PD	Increased AEA, PEA, and OEA levels; reduced L-DOPA-induced side effects Inhibition of dopaminergic neuronal death; decreased microglial immunoreactivity; improved motor capacity	[225] [226]
	URB597 JNJ1661010 TCF2	Chronic mouse model of PD	Anti-cataleptic effects	[227]
	URB597 CAY100400 CAY100402	Experimental autoimmune encephalomyelitis (EAE) in mice	Anti-spastic effects	[228]
MAGL inhibitors	JZL184	LPS-induced neuroinflammation in rats	Decreased peripheral and central cytokine production	[229]
		EAE in mice	Anti-spastic effects	[228]
		Mouse model of AD	Improvements in spatial learning and memory decreased proinflammatory reactions of microglia and reduced amyloid β burden	[230] [231]
		TBI rat model	Improved neurobehavioral recovery; reduce astrocyte activation; less glutamate dyshomeostasis	[232]
	CPD-4645	LPS-induced neuroinflammation and focal photothrombotic ischemic insult in mice	Restored functional homeostasis of the brain vasculature	[175]
	KML29	chronic MPTP/probenecid mouse model of PD	Attenuated striatal dopamine depletion; increase in *Gdnf* expression	[223]
		Ulcerative colitis in humans back pain in humans	Increased PEA levels in colon enhanced PEA blood levels after therapeutic manipulation	[233]
	ABX-1431	Formalin pain model in rats	Increased brain 2-AG concentrations; suppressed pain behavior	[234]
dual MAGL FAAH inhibitors	JZL195	Traumatic brain injury in rats	Attenuated neuronal dysfunction	[235]
NAAA inhibitors	AM9053	Neuropathy in mice	Reversed and prevented peripheral neuropathy via PPAR-*α*	[236]
	Compound 8	EAE in mice	Delayed disease onset; attenuated symptom intensity; normalized body weight; reduced leukocyte infiltration and microglia activation	[237]
	oxazolidin-2-one (F96)	Ear edema model in mice	Prevented allodynia via PPAR-α	[238]
	EPT4900	Carrageenan-induced pain in rats	Inhibited inflammation as well as hyperalgesia	[239]
	ARN077	Rodent models of hyperalgesia and allodynia	Prevented heat hyperalgesia and mechanical allodynia via PPAR-α	[240]

**Table 4:** Effects of CB_1_/CB_2_ receptor activation on leukocyte function.

CB receptor	Compound	Cell Type	Effect	References
CB_1_	AEA ACEA	Monocytes Macrophages	Promotes ROS and cytokine production	[271]
CB_2_	JWH-015	Macrophages	Inhibits ROS production	[271]
	2-AG	Neutrophils	Inhibits migration	[272]
			Promotes migration	[273]
	JWH-133	Neutrophils	Inhibits adhesion to cerebral endothelial cells	[274]
		T cells	Inhibits migration	[275,276]
	2-AG	T cells	Inhibits migration	[276]
CB_1_/CB_2_ independent	2-AG	Neutrophils	Stimulates myeloperoxidase and leukotriene release, kinase activation, and calcium mobilization	[277]
			Release antimicrobial effectors	[278]
